# Advances in laboratory diagnostic methods for cerebrospinal fluid testing for neurosyphilis

**DOI:** 10.3389/fpubh.2022.1030480

**Published:** 2022-11-14

**Authors:** Zheng-Xiang Gao, Yu Gou, Xiao-Qin Liu, Lei-Wen Peng

**Affiliations:** ^1^Department of Laboratory Medicine, West China Second University Hospital, Sichuan University, Chengdu, China; ^2^Key Laboratory of Birth Defects and Related Diseases of Women and Children, Sichuan University, Ministry of Education, Chengdu, China

**Keywords:** *Treponema pallidum*, cerebrospinal fluid, neurosyphilis, testing, syphilis

## Abstract

Neurosyphilis is a chronic infectious disease caused by the invasion of *Treponema pallidum* into the central nervous system. In recent years, with the increase in the latent syphilis infection rate, the incidence of neurosyphilis has gradually increased, the typical symptoms of neurosyphilis have decreased, atypical manifestations have increased, and the clinical manifestations have become increasingly diverse. Cerebrospinal fluid testing plays an important role in the diagnosis of neurosyphilis. In recent years, there have been many advances in cerebrospinal fluid testing. This review focuses on the current and potential laboratory indicators of neurosyphilis in cerebrospinal fluid, aiming to provide a reference for clinical application and ideas for future experimental research of neurosyphilis.

## Introduction

Syphilis is a chronic infectious disease caused by *Treponema pallidum* (*T. pallidum*), and humans are the only host. *T. pallidum* is highly neuroinvasive and can invade the central nervous system and result in neurosyphilis at any time after the initial infection. Studies have shown that *T. pallidum* can disseminate to the central nervous system of rabbits within hours to days after inoculation (1). In recent years, with the increasing prevalence of syphilis around the world, the incidence of neurosyphilis has also increased markedly each year (2).

**Figure 1:**
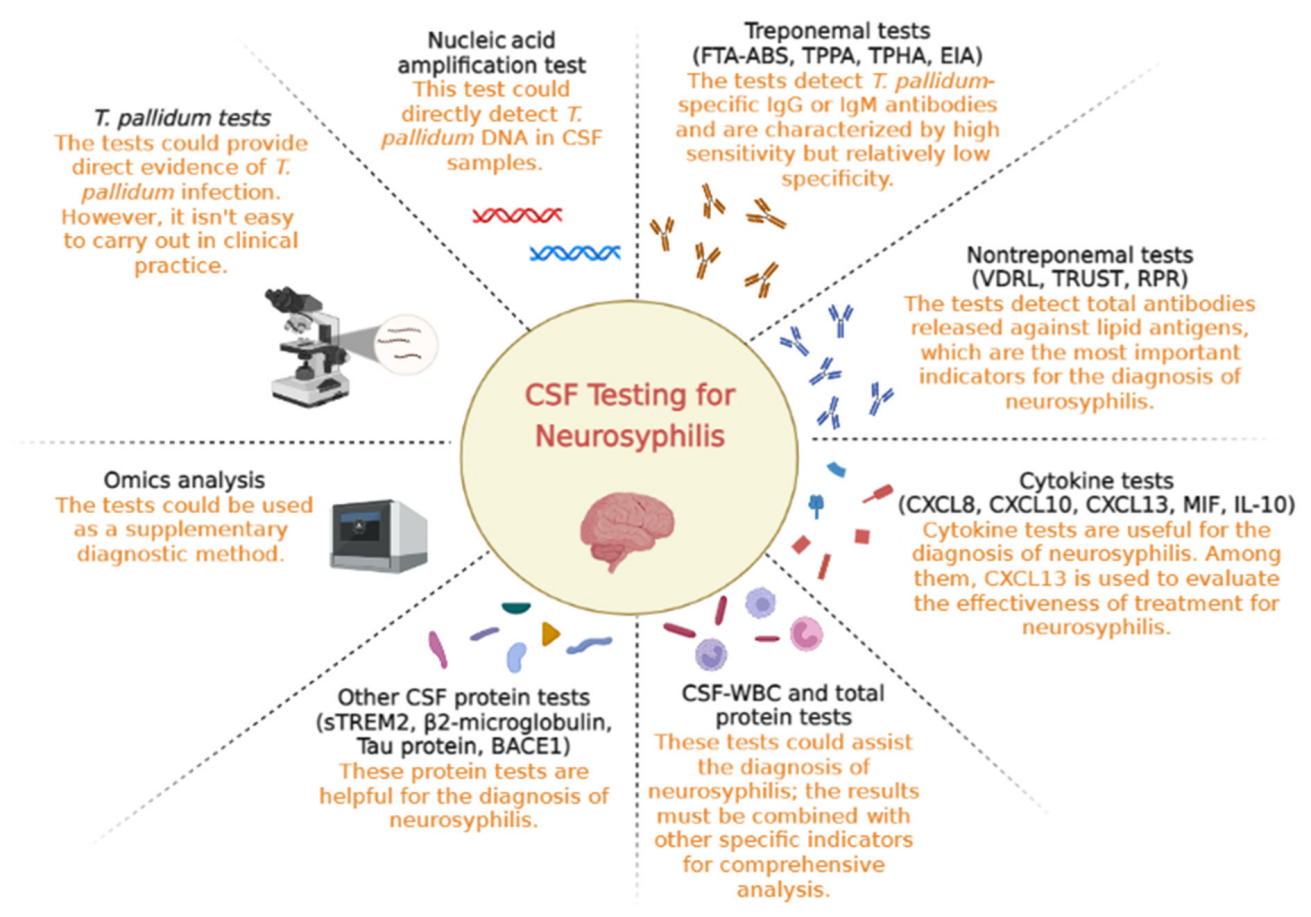
Overview of cerebrospinal fluid testing for neurosyphilis.

Neurosyphilis varies according to the infection period and central site of infection. In the early stage of infection, *T. pallidum* mainly invades meningeal nerves and meningeal blood vessels, which could cause meningitis, cranial nerve damage, multiple nerve root disease and meningeal damage. Meningeal vascular damage mainly manifests as occlusive cerebrovascular syndrome, which can occur as hemiplegia, aphasia, or epileptic seizures. In the late stage of infection, *T. pallidum* further invades the brain parenchyma, resulting in clinical manifestations such as paralytic dementia and spinal tuberculosis. *T. pallidum* can also cause symptoms of optic nerve and auditory nerve damage, which are known as ocular syphilis and ear syphilis (3). There is no clear time point that marks the division between early and late neurosyphilis. Different types of neurosyphilis are manifestations of different time periods of the disease, and there is often partial overlap. The diagnostic model for neurosyphilis has limitations. To date, there is no commonly accepted gold standard test for neurosyphilis diagnosis, and the algorithms used for the diagnosis of neurosyphilis vary between countries. Cerebrospinal fluid (CSF) testing plays a very important role in the diagnosis of neurosyphilis. In recent years, there has been progress in developing new biomarkers for CSF testing, and these biomarkers are helpful for neurosyphilis diagnosis (see Figure 1). This review summarizes the current and potential laboratory indicators in CSF testing to provide a reference for the diagnosis of neurosyphilis.

## Pathological testing for *T. pallidum* in CSF

### Dark-field examination, direct immunofluorescence and silver staining

The three methods of dark-field examination, direct immunofluorescence and silver staining are often used to detect *T. pallidum* in chancroid lesions or regional lymph nodes that are enlarged in syphilis. Because the lesions contain large numbers of treponemes, the positivity rate and specificity are high. However, the volume of CSF that can be used for testing is low, and the number of *T. pallidum* in CSF is low, resulting in the low sensitivity of the methods mentioned above. Therefore, these methods are difficult to use for the clinical diagnosis of neurosyphilis (4).

### Rabbit infection test

Traditionally, the reference method for the direct detection of *T. pallidum* was the rabbit infection test (RIT). First, the CSF of an individual suspected of having neurosyphilis was injected into the testes of normal New Zealand rabbits. One week after inoculation, the testicular size of rabbits was examined, and blood was used for serological testing (*Treponema pallidum* particle agglutination (TPPA) test and rapid plasma response (RPR) test). If the results of the rabbit serological tests were positive, the animal was considered positive. CSF-RIT could provide direct evidence of *T. pallidum* invasion of the nervous system (4). However, due to the time-consuming, expensive and cumbersome nature of CSF-RIT, it is difficult to carry out in clinical practice. With the abuse of antibiotics, CSF-RIT sensitivity has been further reduced, and this method is used only for laboratory research. Tong et al. found that RIT was no longer highly sensitive for the detection of *T. pallidum* in clinical samples and was not considered adequate as a reference method for measuring the sensitivity of other methods in the current era (5). In addition, recent research has revealed the viability of the long-term continuous propagation of *T. pallidum in vitro*. However, this methodology is also cumbersome and time-consuming (6). Therefore, it is undeniable that RIT is useful for recovering *T. pallidum* from infected tissues and maintaining its survival for scientific studies.

### Nucleic acid amplification test

Due to the biological characteristics of *T. pallidum* and the performance restrictions of RIT and dark-field observation, it is difficult to culture *T. pallidum in vitro*. Therefore, microbiological examination of *T. pallidum* is not possible to use for diagnosing neurosyphilis. With the development of science and technology, the detection of pathogen nucleic acids by nucleic acid amplification tests (NAATs) is becoming increasingly important in the detection of infectious disease pathogens. Molecular assays with NAATs of *T. pallidum* DNA are used for direct detection to improve diagnostic sensitivities. There are various NAATs, such as PCR, nested PCR, quantitative PCR, and reverse transcriptase PCR (4). Villanueva et al. found that different storage temperatures and length of time after CSF specimen collection had little effect on the stability of PCR products used to detect *T. pallidum* in CSF. This result indicates that CSF-PCR may have important clinical value in the diagnosis of neurosyphilis (7). Various *T. pallidum* genes have been used for NAATs, including those encoding treponemal surface or subsurface lipoproteins (such as the DNA polymerase I gene [i.e., polA], *T. pallidum* 47 kDa lipoprotein [Tp47], subsurface lipoprotein 4D [4D], treponemal membrane protein A [tmpA] and basic membrane protein [bmp]) (8–10). Among these targets, the most frequently described and evaluated target genes are Tp47 and polA.

Direct detection of *T. pallidum* DNA in CSF by NAATs has been assessed as a potential additional tool for the diagnosis of neurosyphilis. A total of 124 patients with positive *T. pallidum* serological results were recruited and classified according to the 2008 European syphilis management guidelines. Among these patients, Tp47 and polA NAATs were positive in 25 (76%) and 23 (70%) of 33 neurosyphilis patients and 12 (13%) and 7 (8%) of 91 patients without neurosyphilis, respectively (11). Another prospective study evaluated the diagnostic value of nested PCR for the detection of *T. pallidum* in CSF specimens. The nested PCR assay had an overall sensitivity of 42.5%, a specificity of 97%, a positive predictive value of 77%, and a negative predictive value of 86%. These results suggest that PCR is highly specific and has potential value when used together with other neurosyphilis indicators (12).

However, in a recent study, the CSF-PCR results of 8 patients with clinically diagnosed neurosyphilis were negative, suggesting that the clinical application of these methods is still far in the future (13). Another meta-analysis related to CSF-PCR showed that the sensitivity of PCR for the definitive diagnosis of neurosyphilis varied from 40 to 70%, and the specificity ranged from 60 to 100%. Tp47 is the most commonly used target gene in PCR assays, with an overall sensitivity of 68% and a specificity of 91.9%, which were lower than those of the treponemal test (14). Currently, the use of a CSF-PCR assay to detect *T. pallidum* DNA requires further study. The low detection rate might be because *T. pallidum* is less abundant in the CSF of neurosyphilis patients. On the one hand, the time and clinical features of *T. pallidum* invasion into the nervous system should be explored. On the other hand, methods to improve the DNA detection rate in specimens with low *T. pallidum* loads should be studied. Recent studies have shown that digital PCR, detecting trace genes, can detect *T. pallidum* DNA in saliva, and there is no significant difference in the detection rate between saliva and plasma. Whether this technique could be used for the detection of *T. pallidum* DNA in CSF remains to be further studied (15).

## Non-treponemal tests

Non-treponemal tests detect total antibodies (IgM and IgG) released against lipid antigens (e.g., cardiolipin and lecithin) and non-specific antibodies produced by *T. pallidum* surface lipids after *T. pallidum* infection of host cells. The non-treponemal tests mainly include the venereal disease research laboratory (VDRL) test, toluidine red unheated serum test (TRUST) and RPR test (16). The measuring principles and processes of these tests, which involve quantitative detection through continuous dilution of serum, are similar, and the results are reported by titer. These three tests could be used for CSF detection in neurosyphilis patients.

The CSF-VDRL test has been considered the gold standard for neurosyphilis diagnosis for a long time and is the main diagnostic test for neurosyphilis in domestic and foreign guidelines (17–21). It is generally believed that the CSF-VDRL test has high specificity but low sensitivity for the diagnosis of neurosyphilis. A meta-analysis showed that the sensitivity of the CSF-VDRL test was 49–87%, and the specificity was 74–100% for diagnosis (22). The proportion of positive CSF-VDRL tests was higher in neurosyphilis patients who were infected by human immunodeficiency virus (HIV) (23). Performing the VDRL test is complicated; the reagent must be prepared before testing, the specimen must be tested within 2 h after collection, and the results are observable only by optical microscopy. Furthermore, other diseases might influence VDRL results. A case report described a false-positive CSF-VDRL test result in a patient with central nervous system malignancies (24). CSF contamination by blood (which may occur in situations such as craniocerebral trauma) may lead to false-positive CSF-VDRL test results in patients with syphilis (25).

The RPR test uses the same antigen as the VDRL test except it is bound to carbon particles and uses charcoal particles as the visualizing agent. Studies have reported that the CSF-RPR test sensitivity ranged from 51.5 to 81.8%, and the specificity ranged from 81.8 to 100% (26, 27). The TRUST is similar to the RPR test but uses toluidine red as the visualizing agent rather than charcoal. It has been reported that CSF-TRUST sensitivity ranges from 58.9 to 82.5%, and specificity ranges from 82.1 to 93.1% (27). The operation of the RPR test and TRUST is simple, and the results are stable and can be observed by the naked eye without a microscope.

One study focused on the performance of a non-treponemal test in CSF neurosyphilis specimens and found that the sensitivity and specificity of the CSF-VDRL test, CSF-RPR test and CSF-TRUST were similar. Therefore, the CSF-RPR test and CSF-TRUST could be considered alternative tests for neurosyphilis in HIV-negative patients, especially when the CSF-VDRL test is unavailable (27). However, another study showed that CSF-RPR test sensitivity and specificity are lower than those of the CSF-VDRL test (26).

At present, there is no unified view on the sensitivity and specificity of the CSF-VDRL test, CSF-RPR test and CSF-TRUST, and it is unlikely that the role of the CSF-VDRL test could be completely replaced by the CSF-RPR test and CSF-TRUST in the diagnosis of neurosyphilis. There are also some different opinions about the role of CSF non-treponemal tests in the diagnosis of neurosyphilis among different country guidelines. The guidelines for the Treatment of Sexually Transmitted Diseases of the United States Centers for Disease Control and Prevention (CDC) emphasize that CSF-VDRL test positivity could be used as an important diagnostic criterion for symptomatic neurosyphilis patients, but CSF-VDRL test negativity does not exclude a neurosyphilis diagnosis (28). The European Syphilis Management Guidelines suggest that CSF-VDRL/RPR test positivity could diagnose neurosyphilis (20). CSF-VDRL test abnormalities have been included in the diagnostic criteria of neurosyphilis in the Chinese Guidelines for Diagnosis and Treatment of Syphilis. The Chinese and European Guidelines pointed out that the CSF-TRUST or CSF-RPR test could be used to replace the CSF-VDRL test in the absence of these conditions (17, 20).

CSF non-treponemal test results are the most important indicators for the diagnosis of neurosyphilis at present. However, this test is difficult to automate, the results observed by the naked eye are relatively subjective, and the sensitivity is not high. Biological false positives due to cross reactivity may be related to various infectious and non-infectious diseases, and the anterior band phenomenon may cause false-negative results. Therefore, it is necessary to determine more sensitive laboratory indicators as supplementary tests for CSF non-treponemal tests.

## Treponemal tests

Treponemal tests use *T. pallidum* extract or recombinant *T. pallidum* protein as a specific antigen to detect serum anti-*T. pallidum* IgG or IgM antibodies. At present, treponemal tests mainly include the fluorescent treponemal antibody absorption (FTA-ABS) test, *Treponema pallidum* haemagglutination assay (TPHA), TPPA test and enzyme immunoassay (EIA). The FTA-ABS, TPHA and TPPA tests are used to detect total antibodies against *T. pallidum* antigen. EIA mainly detects IgG and IgM antibodies against recombinant *T. pallidum* proteins (such as TpN15, TpN17 and TpN47). The tests mentioned above can be used to detect anti-*T. pallidum* IgG or IgM antibodies in CSF. Studies have reported that the sensitivity of the CSF-FTA-ABS test is 90.9–100%, and the specificity is 100% (29–31). It has been reported that the CSF-TPPA test sensitivity is 75.6–95%, and the specificity ranges from 85.5 to 100% (31–33). One study showed that the diagnostic specificity of a CSF-TPPA titer ≥1:640 was significantly higher than that of lower dilutions and was not significantly different from that of the CSF-VDRL test (31). There was no significant difference in CSF-TPPA test results between HIV-infected and non-infected neurosyphilis patients (31). The TPHA test is similar to the TPPA test, and a study has shown that a CSF-TPHA titer ≥1:640 is specific for the diagnosis of neurosyphilis (34). The CSF-FTA-ABS test requires fluorescence microscope observation, while the TPHA and TPPA tests are simpler flocculation tests, and the results can be observed by the naked eye.

One study revealed the performance of EIA tests in detecting CSF specimens. The Trep-Sure and Maxi-Syph tests measure both IgG and IgM anti-treponemal antibodies. The sensitivity of Trep-Sure was 92.9%, the sensitivity of Maxi-Syph was 100%, and the specificity of both tests was 100% (32). Although the results showed high sensitivity and specificity of the CSF-EIA test in the diagnosis of neurosyphilis, it is worth noting that there were few samples, and the data in this study were limited. In addition, a systematic review of CSF treponemal tests for neurosyphilis indicated that there were significant differences in the sensitivity and specificity of treponemal tests in different studies (35).

Because the anti-treponemal IgG antibody can cross the blood–brain barrier and enter the CSF, which causes false positives in treponemal tests, it is considered to be a controversial indicator in the diagnosis of neurosyphilis (33). Therefore, the diagnosis of neurosyphilis could be excluded in syphilis patients with negative treponemal tests according to the guidelines for the diagnosis and treatment of syphilis in the United States and Europe. The Chinese guideline takes CSF-FTA-ABS test results as a diagnostic indicator of neurosyphilis and points out that the CSF-TPPA test could be used to replace the CSF-FTA-ABS test in the absence of these conditions. The CSF treponemal test plays an important role in the diagnosis of neurosyphilis, and it is characterized by high sensitivity but relatively low specificity. The positive results need to be evaluated along with clinical manifestations, such as the CSF-VDRL test results and the CSF white blood cell count and protein quantity, to better diagnose neurosyphilis.

## Cytokine tests

### Chemotactic factor tests

Chemokines are small cytokines or signaling proteins secreted by cells. Chemokines and their receptors are expressed in the neurons and glial cells of the central nervous system. Increased levels of chemokines in CSF were found to be associated with the severity or progression of inflammatory diseases of the central nervous system. Chemokines are the most studied cytokines as potential CSF indicators in neurosyphilis patients, among which CXCL13 has been studied extensively and intensively. The concentrations of CXCL13, CXCL10, and CXCL8 were increased in the CSF of neurosyphilis patients, which was related to the CSF protein concentration and the CSF-VDRL titer. The sensitivity/specificity of CXCL13, CXCL10, and CXCL8 in the diagnosis of neurosyphilis were 85.4/89.1%, 79/90.1%, and 79.6/91.1%, respectively (36). Furthermore, there was no correlation between serum and CSF concentrations of CXCL13, which means that the increased CSF CXCL13 level is the result of enhanced synthesis of nervous system cells (37). Some studies have defined asymptomatic neurosyphilis as CSF-VDRL positivity or CSF WBC count > 20 × 10^6^/L and defined symptomatic neurosyphilis as visual or hearing loss. It was found that CSF CXCL13 ≥250 pg/mL had a sensitivity of 41% for both asymptomatic and symptomatic neurosyphilis and a specificity of 93 and 79%, respectively (38). Another study defined neurosyphilis as CSF-VDRL or CSF-TRUST positive results and CSF WBC count >10 × 10^6^/L and/or protein level higher than 500 mg/L. The results showed that the diagnostic sensitivity of CSF CXCL13 concentration ≥13.37 pg/mL in neurosyphilis patients was 84.9%, and the specificity was 78.87% (39). HIV infection can slightly increase CSF CXCL13 concentration, which is negligible in the case of a large increase in CSF CXCL13 concentration in neurosyphilis patients (40). Another study indicated that CSF CXCL13 is more suitable for the diagnosis of neurosyphilis patients with HIV infection (41). At present, CSF CXCL13 is considered to be a potential indicator of the antibiotic treatment response and is used to evaluate the effectiveness of treatment for neurosyphilis (39). However, it is noteworthy that the sensitivity and specificity of CSF CXCL13 are different in neurosyphilis due to the different diagnostic criteria, population characteristics and cut-off values of CSF CXCL13. Further studies are needed to support the diagnostic value of CSF CXCL13.

### Macrophage migration inhibitory factor test

Macrophage migration inhibitory factor (MIF) is a multieffect immunoregulatory cytokine with a unique structure. MIF plays an important role in innate immunity, immune cell recruitment and inflammation. Therefore, it has become a relevant indicator of the inflammatory response in various diseases. MIF is a key indicator of central nervous system infection. Studies have shown that the sensitivity of CSF MIF concentration for neurosyphilis diagnosis was 74.42%, and the specificity was 67.74% (42). The MIF level was higher in the CSF of neurosyphilis patients than in syphilis/non-neurosyphilis patients. MIF has been considered a new potential CSF indicator to establish or exclude the diagnosis of neurosyphilis, but further study is needed.

### Interleukins

Interleukins are cytokines produced by multiple cells that act on multiple cells. Interleukins play an important role in immune function modulation. Studies have shown that the level of IL-17A in the CSF of patients with asymptomatic neurosyphilis is significantly higher than that in patients without neurosyphilis (43). In another study, researchers detected changes in IL-1β, IL-4, IL-6, IL-10, IL-17A and IL-21 levels in the CSF of neurosyphilis patients before penicillin treatment. The results showed that only IL-10 was significantly increased. The sensitivity/specificity of CSF IL-10 in neurosyphilis and asymptomatic neurosyphilis were 86.7/91.7% and 83.3/91.7%, respectively. It is suggested that the CSF IL-10 concentration is useful for the diagnosis of neurosyphilis, especially for asymptomatic neurosyphilis patients. Furthermore, it was found that the CSF IL-10 level was positively correlated with the level of neuronal damage markers, the CSF protein concentration, the CSF white blood cell count and the CSF-RPR titer in neurosyphilis patients. These results suggested that excessive CSF IL-10 might promote the further development of neurosyphilis (44). However, the number of patients included in the above two studies was small. The diagnosis of neurosyphilis with interleukins as a single indicator lacks specificity, and the various interleukins can be elevated in intracranial infection. Therefore, it is necessary to combine CSF indicators for neurosyphilis diagnosis.

## CSF white blood cells and total protein tests

The CSF white blood cell (WBC) count and total protein test, as traditional laboratory indicators for the diagnosis of central nervous system infection, also play an important role in the diagnosis of neurosyphilis. Abnormalities in the routine biochemical examination of CSF are considered important laboratory evidence for the diagnosis of neurosyphilis in many national guidelines. The European guidelines for the diagnosis and treatment of neurosyphilis specifically suggest that CSF examination must include total protein, monocyte count, and treponemal and non-treponemal tests. If there is an abnormal CSF examination result (high protein levels and/or increased number of cells), CSF analysis must be repeated after treatment (6 weeks-6 months) (45). The American guidelines suggest that neurosyphilis should also be considered if the CSF-VDRL result is negative but the patient has neurological symptoms and/or signs, positive serological tests for syphilis, and abnormal CSF white blood cell count and/or protein levels. If neurosyphilis patients are coinfected with HIV, CSF WBC counts are usually elevated (>5 × 10^6^/L), with >20 × 10^6^/L used as the cut-off value to improve the specificity of neurosyphilis diagnosis (19). In the Chinese guidelines, neurosyphilis can be diagnosed in syphilis patients who meet the following two criteria: (1) CSF white blood cell count ≥5 × 10^6^/L (combined with HIV infection, white blood cell count is often >20 × 10^6^/L) and protein > 500 mg/L, with no other evident causes of these abnormalities. (2) Positive CSF-VDRL test (or RPR test/TRUST) or FTA-ABS test (or TPPA/TPHA test) (17). Another study has shown that the more WBCs there are in the CSF of neurosyphilis patients, the higher the total protein level and the worse the prognosis, suggesting that CSF WBCs and total protein are of great significance in judging the progress of neurosyphilis (46). However, CSF WBC count and total protein level lack specificity. The protein levels and the number of monocytes in the CSF of neurosyphilis patients may be normal, and a large number of monocytes in the CSF could be observed in many cases, including HIV infection without syphilis. Therefore, CSF WBC count and total protein level can only assist the diagnosis of neurosyphilis, and the results must be combined with other specific indicators for a comprehensive analysis.

## Other CSF protein tests

### Tau protein

Tau protein is the main microtubule-associated protein in neurons and is located mostly in the axons of neurons. A study found that there was a significant difference in CSF Tau protein content between neurosyphilis and Alzheimer's disease patients. The Tau protein level in the CSF of neurosyphilis patients was significantly higher than that of non-neurosyphilis patients, suggesting that Tau protein is helpful for the diagnosis of neurosyphilis with a history of syphilis and cognitive decline (47, 48). However, Tau protein is an acute-phase reactive protein that is increased to different degrees in a variety of neurological diseases, especially neurodegenerative diseases, with low specificity (49). Whether Tau protein can be an indicator of neurosyphilis needs further study.

### β-amyloid precursor protein lyase

β-Amyloid precursor protein lyase (BACE1) is a key enzyme responsible for the cleavage of pathological amyloid β-protein precursors (50). The study found that plasma BACE1 levels were significantly correlated with CSF BACE1 levels in the neurosyphilis group. Therefore, plasma BACE1 may be a promising biomarker for early diagnosis (48).

### Triggering receptor expressed on bone marrow cells 2

Triggering receptor expressed on bone marrow cells 2 (TREM2) is a cell surface receptor protein. For the brain, the focus is on the expression of TREM2 in microglia, which promotes phagocytosis, inhibits toll-like receptor-induced inflammatory cytokine production, and enhances anti-inflammatory cytokine transcription *in vitro* (51, 52). Its soluble variant (sTREM2) can be detected in CSF. Studies have found that the level of CSF sTREM2 was significantly higher in neurosyphilis patients than in syphilis/non-neurosyphilis patients, and sTREM2 levels were higher in late neurosyphilis than in early neurosyphilis, suggesting that sTREM2 can provide a basis for the clinical prognosis of neurosyphilis (53).

### β2-microglobulin

β2-microglobulin is a protein composed of 11 polypeptide chains that is one of the light chain components of major histocompatibility complex class I molecules and is associated with aging-related cognitive impairment and Alzheimer's disease (54). The level of β2-microglobulin in normal CSF is very low. If the concentration of β2-microglobulin in CSF increases, it indicates that there is pathological damage in the central nervous system. Studies have found that the expression of β2-microglobulin in the CSF of children with congenital syphilis increases significantly, and the level of β2-microglobulin gradually decreases to normal after corresponding treatment. These results suggest that β2-microglobulin is very useful in the diagnosis of congenital syphilis central nervous system injury and in monitoring the response to treatment (55).

## Omics analysis

### CSF metagenome sequencing analysis

Increasing data have shown that metagenomic sequencing can play a role in neurological infectious diseases (such as unexplained encephalitis and meningitis), and metagenomic next-generation sequencing (mNGS) analysis of CSF can quickly detect pathogens. A recent study showed that *T. pallidum* in CSF was detectable by metagenomic sequencing. However, it is difficult to use metagenomic sequencing as a conventional screening tool due to its high cost. For neurosyphilis patients with atypical symptoms or negative results as detected by conventional methods, mNGS could be used as a supplementary diagnostic method.

### CSF metabolomics analysis

Metabolomics is a quantitative analysis of all metabolites in organisms and a search for the relative relationship between metabolites and physiological and pathological changes. Metabolomics provides a large amount of information on energy metabolism, physiology and possible pathogen diagnostic biomarkers and intervention strategies. A metabolic analysis of CSF in patients with neurosyphilis showed that bilirubin, L-histidine, prostaglandin E2, α-mannuronic acid, butyryl L-carnitine and palmitoyl L-carnitine were significantly increased in the CSF of neurosyphilis patients. The metabolism of CSF may provide a new way to explore the pathogenesis of neurosyphilis (56). In another study, several metabolites in the CSF of neurosyphilis patients were found to be significantly altered, including d-mannitol, N-acetyl-tyrosine, hypoxanthine and s-methyl-5'-adenosine, and N-acetyl-tyrosine was 87.4 and 7.5 times higher in the CSF of neurosyphilis patients than in that of non-secondary syphilis patients and non-syphilis patients, respectively. However, the metabolites in the CSF of neurosyphilis patients lack specificity, and the types of metabolites reported in different studies are not consistent. Whether these metabolites can be new potential markers of neurosyphilis needs further evaluation and study (57).

## Conclusions

At present, the diagnosis of neurosyphilis is mainly based on the patient's epidemiological history, clinical manifestations and laboratory tests. Elevated CSF WBC count and total protein level are an important basis for suspected neurosyphilis, but the existing diagnosis cannot meet clinical needs. The sensitivity and specificity of non-treponemal and treponemal tests are different. Because the existing laboratory tests cannot meet clinical needs, the diagnosis of neurosyphilis still faces challenges. With the development of related technologies, new methods and biomarkers have emerged for CSF detection in neurosyphilis. Detecting *T. pallidum* DNA in CSF by NAATs has gradually become possible, and CSF cytokines as indicators for neurosyphilis diagnosis continue to be defined. CSF CXCL13, which shows good diagnostic efficacy, is most likely to be applied in clinical practice. Interleukin, MIF, Tau protein, BACE1, sTREM2, and β2-microglobulin and metagenomic and metabolomic factors are considered to be potential markers in the CSF detection of neurosyphilis. However, the specificity of these indicators is not high, and the related studies are few; therefore, further study is needed (see Table 1). In conclusion, no single test can diagnose all cases of neurosyphilis. Generally, the diagnosis of neurosyphilis is based on clinical manifestations and CSF tests. The CSF tests should include non-treponemal and treponemal tests and CSF white blood cell count and total protein level tests. If necessary, NAATs, cytokine tests and other CSF protein tests should be performed to assist the diagnosis of neurosyphilis. Furthermore, new diagnostic markers with high sensitivity and specificity are needed. Carrying out high-quality multicentre clinical research is very important for future neurosyphilis laboratory diagnostic Studies.

**Table 1:** Summary of the performance characteristics for the different CSF tests in neurosyphilis.

**Test**		**Sensitivity (%)**	**Specificity (%)**	**Characteristics**	**References**
Dark-field examination Direct immunofluorescence Silver staining		–	–	The number of *T. pallidum* in CSF is low, resulting in the low sensitivity of these methods. Therefore, the methods are difficult to use for the clinical diagnosis of neurosyphilis.	(4)
Rabbit infection test (RIT)		–	–	The CSF-RIT can provide direct evidence of *T. pallidum* invasion of the nervous system. However, the CSF-RIT is difficult to carry out in clinical practice.	(4)
Nucleic acid amplification test (NAAT)		40–70	60–100	The NAAT enables direct detection of *T. pallidum* DNA in CSF. Its sensitivity and specificity are lower than those of treponemal tests, and further study is needed.	(11, 12, 14)
Non-treponemal tests	VDRL	49–87.5	74–100	CSF non-treponemal test results are the most important indicator for the diagnosis of neurosyphilis at present. However, these tests are difficult to automate, the results observed by the naked eye are relatively subjective and the sensitivity is not high. Many diseases can cause biological false-positive non-treponemal test results.	(22, 27, 31)
	RPR	51.5–81.8	81.8–100		(26, 27)
	TRUST	58.9–82.5	82.1–93.1		(27)
Treponemal tests	FTA–ABS	90.9–100	55–100	*T. pallidum* IgG can cross the blood–brain barrier, which can cause false-positive treponemal test results. Positive results need to be evaluated along with the clinical manifestations, CSF-VDRL test results, CSF white blood cell count and protein quantity to better diagnose neurosyphilis.	(29–31)
	TPPA	75.6–95	85.5–100		(31–33)
	EIA	92.9–100	100		(32)
Cytokine tests	CXCL13	84.9–85.4	78.87–89.1	Chemokines are increased in the CSF of neurosyphilis patients and are not influenced by serum concentrations. Among the various cytokines, CXCL13 has been considered to be a potential indicator of the effect of antibiotic treatment in neurosyphilis.	(36–41)
	CXCL10	79	90.1		(36)
	CXCL8	79.6	91.1		(36)
	MIF	74.42	67.74	MIF concentration can be considered as a new potential CSF indicator to establish or exclude the diagnosis of neurosyphilis, but further studies are needed.	(42)
	IL-10	83.3–86.7	91.7	CSF IL-10 concentration is useful for the diagnosis of neurosyphilis, especially for asymptomatic neurosyphilis patients. The diagnosis of neurosyphilis with interleukin as a single indicator lacks specificity.	(44)
CSF white blood cell count and total protein level tests		–	–	CSF WBC count and total protein level can only assist the diagnosis of neurosyphilis; the results must be combined with other specific indicators for comprehensive analysis.	(17, 19, 45, 46)
Other CSF protein level tests (sTREM2, β2-microglobulin, Tau protein and BACE1)		–	–	The specificity of these indicators is not high, and the related studies are few; therefore, further study is needed.	(47–55)
CSF mNGS analysis		–	–	The cost is expensive, and the test is difficult to use as a conventional screening tool. mNGS can be used as a supplementary diagnostic method.	(58)
CSF metabolomics analysis		–	–	Metabolites in the CSF of neurosyphilis patients lack specificity, and the types of metabolites investigated in different studies are not consistent.	(56, 57)

## Author contributions

L-WP designed and edited the manuscript. Z-XG wrote the paper. YG and X-QL collected and analyzed the data. All authors contributed to the article and approved the submitted version.

## Funding

This work was supported by the Foundation (grant number: 21H1219). The funders played no role in the study design, data collection and analyses, decision to publish, or manuscript preparation.

## Conflict of interest

The authors declare that the research was conducted in the absence of any commercial or financial relationships that could be construed as a potential conflict of interest.

## Publisher's note

All claims expressed in this article are solely those of the authors and do not necessarily represent those of their affiliated organizations, or those of the publisher, the editors and the reviewers. Any product that may be evaluated in this article, or claim that may be made by its manufacturer, is not guaranteed or endorsed by the publisher.
